# β-Lapachone-Induced Oxidative Stress Causes PARP-Dependent NAD^+^-Depletion that Affects the Energy Metabolism of Cultured Primary Rat Astrocytes

**DOI:** 10.1007/s11064-026-04814-7

**Published:** 2026-06-12

**Authors:** Johanna Elisabeth Willker, Ralf Dringen

**Affiliations:** 1 Centre for Biomolecular Interactions Bremen, Faculty 2 (Biology/Chemistry), University of Bremen, P.O. Box 330440, 28334 Bremen, Germany; 2 Centre for Environmental Research and Sustainable Technologies, University of Bremen, Bremen, Germany

**Keywords:** β-Lapachone, Astrocytes, ATP, Glutathione, Nicotinamide coenzymes, Oxidative stress, PARP

## Abstract

Oxidative stress has been connected with many brain pathologies. As brain astrocytes have a strong antioxidative potential, we have investigated the consequences of β-lapachone-induced oxidative stress on the cell metabolism of cultured astrocytes by determining the cellular levels of important components of the cellular redox and energy metabolism. β-Lapachone exposure induced a rapid oxidation of cellular NADH and NADPH followed by a time- and concentration-dependent loss in the total cellular NADx content and an increase in the total cellular NADPx content, while the cell viability was not compromised. In addition, the treated cells were partially depleted of ATP and lost their ability to upregulate glycolytic lactate production after exposure to the respiratory chain inhibitor antimycin A. All these consequences were prevented in the presence of ES936 which inhibits the NQO1-mediated reduction of β-lapachone. Inhibition of poly(ADP-ribose) polymerases (PARPs) by PJ34 or AZD-2461 did not affect the strong cellular accumulation of glutathione disulfide, but significantly lowered the oxidative stress-induced loss in the cellular NADx and ATP contents, maintained the ability of the cells to upregulate glycolytic lactate production during incubation with antimycin A, and doubled the increase in the cellular NADPx content. After removal of the β-lapachone-induced oxidative stress, astrocytes were able to restore their initial cellular content of NADx in the presence of the NAD^+^ precursor nicotinamide. This restoration was accompanied by the reestablishment of the ability to upregulate glycolytic lactate production during antimycin A exposure. The results obtained show that oxidative stress has severe consequences on various fundamental metabolic parameters of astrocytes, but also demonstrate the high potential of these cells to recover after severe oxidative stress.

## Introduction

Oxidative stress and alterations in the energy metabolism in the brain have frequently been connected with acute neuropathologies, such as traumatic brain injury and stroke [1–5], but also with neurodegenerative disorders, including Alzheimer’s and Parkinson’s disease [6–10]. Three cellular redox pairs are crucial for an efficient protection of brain cells against the adverse consequences connected with oxidative stress and for the electron transfer reactions that are essential for ATP regeneration. These are the glutathione redox pair (GSx, consisting of the reduced glutathione (GSH) and the oxidized glutathione disulfide (GSSG)), the nicotinamide adenine dinucleotide phosphate redox pair (NADPx, consisting of the reduced NADPH and the oxidized NADP^+^) and the nicotinamide adenine dinucleotide redox pair (NADx, consisting of the reduced NADH and the oxidized NAD^+^) [11–18].

Reactive oxygen species that are produced in cells during normal metabolism are rapidly removed by cellular GSH-dependent reduction which leads to the generation of GSSG. To maintain a high cellular ratio of GSH to GSSG, GSH is efficiently regenerated by the NADPH-dependent reaction catalysed by glutathione reductase [11]. This enzyme connects the two redox pairs GSx and NADPx which are both essential for efficient antioxidative defence. In contrast, the NADx redox pair has a main cellular function as electron acceptor and donor during catabolic processes that are connected with cellular ATP regeneration [17–19], including cytosolic glycolysis and mitochondrial oxidative phosphorylation. One of the multiple additional functions of NAD^+^ is to serve as substrate for poly(ADP-ribose) polymerases (PARPs), which are activated during oxidative DNA damage and have central functions in DNA repair and cellular regulation [20–22].

The only known cellular reaction that generates NADP^+^ is the phosphorylation of NAD^+^ by NAD kinase (NADK) [23]. This enzymatic reaction connects the NADx redox pair with the NADPx redox pair. The profound interplay between the cellular GSx, NADPx and NADx redox pairs, which was also recently shown for cultured brain astrocytes [16], suggests that oxidative stress can strongly affect the energy metabolism and ATP regeneration in brain cells by altering the contents and the oxidation states of the three cellular redox pairs.

Astrocytes are important partners of neurons and have multiple important supporting and protective functions in the brain [24–28]. Especially concerning brain metabolism, a strong interaction between astrocytes and neurons has been reported [29–33]. Astrocytes have a broad metabolic potential that has been extensively studied using cultured astrocytes as model systems, especially for the GSH [11] and ATP metabolism [34].

β-Lapachone is a natural quinone that is produced in the lapacho tree [35, 36] and has been considered as potential antitumor drug [35, 37–39]. In cells, β-lapachone is reduced to the unstable β-lapachol by the enzyme NAD(P)H: quinone acceptor oxidoreductase 1 (NQO1) [39]. The autoxidation of β-lapachol to β-lapachone via β-lapachone-semiquinone recycles β-lapachone, thereby resulting in the formation of two superoxide molecules per cycle [39]. Application of β-lapachone to cultured astrocytes has been shown to cause NQO1-dependent superoxide production and severe oxidation of cellular GSH to GSSG [40, 41]. The electrons needed by astrocytic NQO1 for the reduction of β-lapachone are mainly provided by NADPH that is regenerated by the pentose-phosphate pathway (PPP) [41, 42]. As both β-lapachone and β-lapachol are membrane permeable and act as redox cyclers [41], these compounds can be efficiently removed from cells by washing [40]. In addition, the superoxide formation and the oxidative stress generated by β-lapachone exposure can be completely prevented by the application of the NQO1 inhibitors dicoumarol or ES936 [40–42]. Therefore, β-lapachone exposure appears to be a suitable experimental paradigm to study the direct consequences of oxidative stress on the cellular metabolism of cultured astrocytes, as well as the recovery of the cells from the adverse effects of oxidative stress.

For cultured astrocytes we have recently shown that peroxide-induced oxidative stress causes rapid GSH oxidation that is followed by a NADK-mediated increase in the cellular NADPx content on the expense of the NADx content [16]. We have now extended our studies on the interplay between the three redox pairs, GSx, NADPx, and NADx, during oxidative stress by investigating the consequences of β-lapachone-induced oxidative stress on these cellular redox pairs as well as on the basal energy metabolism of cultured astrocytes. In addition, we have studied the recovery of astrocytes after a period of oxidative stress caused by β-lapachone. The main new results presented in this study demonstrate that PARP-mediated NAD^+^ consumption during β-lapachone-induced oxidative stress causes a depletion of the cellular NAD^+^ content that subsequently limits energy metabolism. In addition, we show that cultured astrocytes have the potential to fully recover from a short period of extensive oxidative stress. These data provide new insights on the interplay between the different main redox pairs during and after oxidative stress and on following consequences on the astrocytic metabolism but also demonstrate the high potential of astrocytes to recover after severe oxidative stress.

## Methods

### Materials

Antimycin A, 5,5’-dithiobis (2-nitrobenzoic acid) (DTNB), glucose-6-phosphate, glutathione reductase, GSSG, magnesium chloride, 3-(4,5-dimethyl 2-thiazolyl)−2,5-diphenyl-2 H-tetrazolium bromide (MTT) and the redox cyclers phenazine ethosulfate (PES) and phenazine methosulfate (PMS) were purchased from Sigma-Aldrich (Steinheim, Germany). DMEM (containing 25 mM glucose), fetal calf serum (FCS) and penicillin G/streptomycin sulfate solution were obtained from Thermo Fisher Scientific (Schwerte, Germany). Dimethyl sulfoxide (DMSO), NADH, NADPH and sulfosalicylic acid were bought from AppliChem (Darmstadt, Germany). ATP, yeast glucose-6-phosphate dehydrogenase (G6PDH), glutamate-pyruvate transaminase and lactate dehydrogenase (LDH) were purchased from Roche Diagnostics (Mannheim, Germany). Nicotinamide and 2-vinylpyridine were from Fluka (Steinheim, Germany). The Cell Titer Glo^®^ 2.0 ATP Assay Kit was from Promega (Walldorf, Germany). ES936 was obtained from Santa Cruz (Heidelberg, Germany). AZD-2461 and PJ34 were purchased from MedChemExpress (Monmouth Junction, NJ, USA). β-Lapachone (ab141097) was from abcam (Berlin, Germany). All other basal chemicals were purchased from Sigma-Aldrich (Steinheim, Germany), Roth (Karlsruhe, Germany) or Merck (Darmstadt, Germany). Sterile cell culture consumables and unsterile 96-well plates were obtained from Sarstedt (Nümbrecht, Germany).

### Primary Astrocyte Rich Cultures

All experiments of this study were performed on astrocyte-rich primary cultures prepared from the whole brains of newborn Wistar rats as described before [43]. Adult rats had been obtained from Charles River Germany (Sulzfeld, Germany) and were treated in accordance with the animal welfare acts of the State of Bremen, Germany and Europe. The harvested cells were plated in a density of 300,000 cells per well in wells of 24-well plates in 1 mL culture medium (90% DMEM containing 25 mM glucose, 44.6 mM sodium bicarbonate, 1 mM pyruvate, 20 U/mL penicillin G, 20 µg/mL streptomycin sulfate, supplemented with 10% FCS). The primary astrocytes were cultured in a humidified atmosphere with 10% CO_2_ at 37 °C in a Sanyo (Osaka, Japan) CO_2_ cell incubator. The medium was renewed every 7 days and on the day before the experiment. For all experiments performed, confluent astrocyte cultures aged 19–23 days were used. These cultures contain mainly astrocytes and only low numbers of microglial cells and oligodendrocytes [44].

### Experimental Incubations

For the induction of oxidative stress, cultured astrocytes were washed twice with 1 mL warm (37 °C) incubation buffer (IB; 20 mM HEPES, 145 mM NaCl, 1.8 mM CaCl_2_, 1 mM MgCl_2_, 5.4 mM KCl, 0.8 mM Na_2_HPO_4_, pH adjusted to 7.4 at 37 °C with NaOH). Then they were incubated with 250 µL incubation medium (IB containing 5 mM D-glucose with β-lapachone in concentrations of up to 15 µM) at 37 °C in a humidified atmosphere of a cell incubator (without CO_2_ supply) for the incubation periods stated in the legends of the figures and tables. β-Lapachone had been applied from a stock solution in DMSO which led to a final concentration of up to 0.3% of DMSO during the incubations. An incubation of astrocytes with this concentration of DMSO for up to 1 h did not significantly alter the determined parameters compared to the respective incubations without DMSO. To test for potential delayed consequences of oxidative stress and for recovery from the stress, the cultures were preincubated for 15 min without or with 15 µM β-lapachone in 250 µL incubation medium (IB with 5 mM D-glucose) that had been supplemented without or with modulators of cellular enzymes, as indicated in the figure legends, at 37 °C in a humidified atmosphere of a cell incubator. Subsequently, the cells were washed twice with IB before the main incubation(s) were performed in 250 µL of the incubation media given in the legends of the respective figures. After the final incubation, the incubation media were harvested and used to determine the extracellular LDH activity as indicator for a potential loss in cell viability. The cells were washed twice with 1 mL cold (4 °C) phosphate-buffered saline (PBS; 10 mM potassium phosphate buffer pH 7.4 containing 150 mM NaCl) and lysed for the quantification of the cellular contents of NADx, NADPx, GSx or ATP.

### Determination of the Cellular NADx and NADPx Contents

The cellular NADx and NADPx contents were determined as described recently [16, 45]. Cell lysis was performed in 0.5 mL extraction buffer (20 mM NaHCO_3_, 100 mM Na_2_CO_3_, 15 mM nicotinamide, 0.05% (w/v) Triton X-100). Cell lysates of two identically treated wells were pooled and then ultrasonicated. Subsequently, the oxidized nicotinamide cofactors were destroyed in aliquot parts of the lysate by a 30 min incubation at 60 °C in the dark [45]. The contents of NAD^+^, NADH and NADx (sum of NAD^+^ plus NADH) were determined by a cycling assay measuring the reduction of MTT in the presence of PMS, lactate and LDH [45]. The contents of NADP^+^, NADPH and NADPx (sum of NADP^+^ plus NADPH) in the lysates were similarly determined by a cycling assay in the presence of PES, glucose-6-phosphate and G6PDH [45]. Both cycling assays are highly specific for NADx or NADPx, respectively. The sum of the specific values for NADx and NADPx is presented as NAD(P)x.

### Determination of the Cellular GSx and GSSG Contents

The cellular GSx (GSH plus 2x GSSG) and GSSG contents were quantified based on the method by Tietze [46] using an adaptation to microtiter plates [47] as described recently [16]. Cell lysis was performed with 250 µL 1% (w/v) sulfosalicylic acid on ice. Cell lysates of two identically treated wells were pooled. These samples were used to determine the cellular GSx contents by an enzymatic cycling assay with a reaction mixture (0.1 M sodium phosphate buffer, 1 mM EDTA, 0.4 mM NADPH, 0.3 mM DTNB, 0.05 U/well glutathione reductase, pH 7.5) leading to the formation of the chromophore thionitrobenzoate. To determine the GSSG content of lysates, the GSH in the samples was removed by a derivatization with 2-vinylpyridine [43] and the GSSG content was then measured as remaining GSx as described above.

### Determination of the Cellular ATP Content

The quantification of the cellular ATP content was performed as described previously [48] for cells that had been lysed with 200 µL 0.5 M perchloric acid. Cell lysates of two identically treated wells were pooled. The cell lysate was diluted with 0.5 M perchloric acid and then neutralised with 2 M KOH. The samples were centrifuged at 12,100 *g* for 5 min and the pH of the supernatant was adjusted by application of 1.4 M Tris-acetate buffer (pH 7.75). The ATP content was then quantified by a luciferin-luciferase-based luminometric assay [49] using the Cell Titer Glo^®^ 2.0 ATP Assay Kit.

### Determination of the Extracellular Lactate Concentration

The concentrations of extracellular lactate were determined using 10 µL of the media samples by applying a coupled enzymatic assay in a microtiter plate as described before [43]. For the quantification, the NADH formed by the oxidation of lactate by LDH was determined by its absorbance at 340 nm. To enable a complete oxidation of lactate, the reaction was coupled to the glutamate-dependent transamination of pyruvate to alanine by glutamate-pyruvate transaminase in an alkaline glutamate buffer [43].

### Cell Viability and Protein Determination

As an indicator of potential toxicity of a given treatment, the extracellular activity of the cytosolic enzyme LDH was determined as described in detail before [43]. The initial cellular protein content per well of untreated cells was determined by the Lowry method [50], using BSA as standard protein. The specific contents of NADx, NADPx, GSx and ATP were calculated by normalizing the determined cellular contents per well to the initial cellular protein content per well of the respective culture.

### Data Analysis and Statistical Analysis

Data are presented as means ± standard deviation (SD). If not stated otherwise, the experiments were performed on three independently prepared rat astrocyte cultures. Normal distribution was confirmed by the Kolomogorov-Smirnov test for data sets derived from more than 5 independent experiments and was assumed for all data sets derived from less than 5 independent experiments. The software GraphPad InStat 3 (GraphPad, Boston, USA) was used for all statistical calculations. Statistical analysis of multiple groups of data was done by ANOVA (followed by the Bonferroni post-hoc test of selected pairs) and the level of significance of differences is indicated by *p < 0.05, **p < 0.01 and ***p < 0.001. For the comparison of two datasets a paired two-tailed t-test was performed and significances of differences are given by ^#^*p* < 0.05, ^##^*p* < 0.01 and ^###^*p* < 0.001 or by ^+^*p* < 0.05, ^++^*p* < 0.01 and ^+++^*p* < 0.001, as indicated in the legends of the figures and tables. *p* ≥ 0.05 was considered as not significant and such results of significance analyses are not indicated in the figures.

## Results

### Effects of β-Lapachone-Induced Oxidative Stress On the Cellular Contents of Nicotinamide Cofactors

**Fig. 1:**
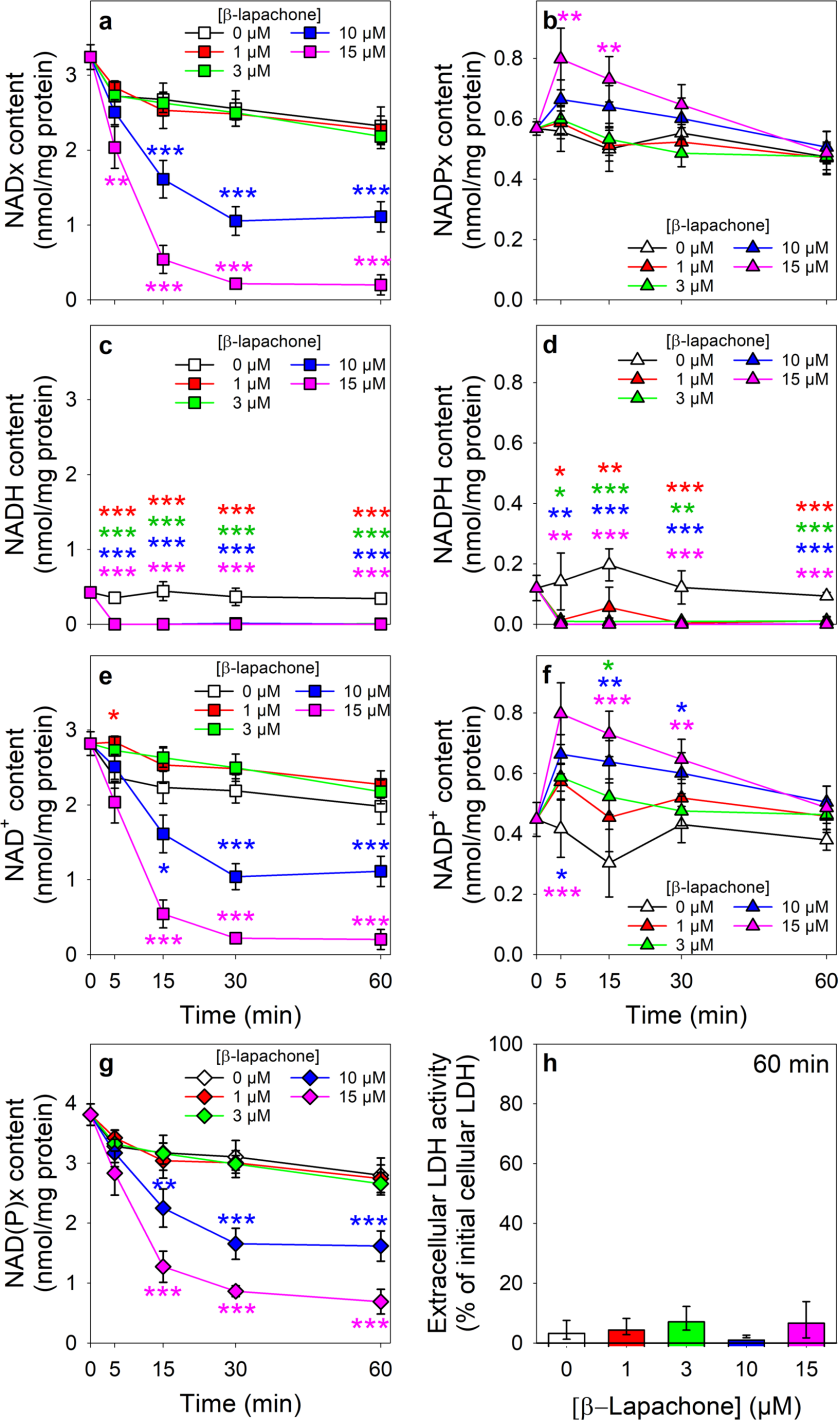
Time- and concentration-dependent alterations in the specific cellular levels of nicotinamide cofactors in cultured astrocytes during incubation with β-lapachone. The cells were incubated in glucose-containing incubation buffer in the absence or the presence of the given concentrations of β-lapachone before the cellular contents of NADx (**a**), NADPx (**b**), NADH (**c**) and NADPH (**d**) were determined. From these data the specific contents of NAD^+^ (**e**), NADP^+^ (**f**) and NAD(P)x (**g**) were calculated. The initial NADx content amounted to 3.24 ± 0.17 nmol/mg protein (of which 87 ± 1% represented NAD^+^) and the initial NADPx content amounted to 0.57 ± 0.02 nmol/mg protein (of which 79 ± 8% represented NADP^+^). The initial cellular LDH activity was 166 ± 26 nmol/(min x well) and the initial protein content of the cultures was 155 ± 7 µg/well. The data shown represent the means ± SD of values determined in three independent experiments performed on independently prepared primary cultures. The significance of differences (ANOVA with Bonferroni post hoc test) compared to the data obtained for the control without β-lapachone (open symbols) is indicated by *p < 0.05, **p < 0.01 and ***p < 0.001 in the colour of the respective condition

Application of β-lapachone-induced oxidative stress to cultured astrocytes has previously been reported to cause rapid and severe oxidation of cellular GSH to GSSG [40]. To investigate whether and how β-lapachone-induced oxidative stress may also affect the cellular levels of the nicotinamide cofactors NADx and NADPx as well as the ratio of oxidized to reduced partners of these redox pairs, β-lapachone was applied to cultured astrocytes in concentrations of up to 15 µM for up to 60 min. An incubation of the cells in the absence of β-lapachone hardly affected the cellular contents of NADx (Fig. 1a), NADPx (Fig. 1b) and NAD(P)x (Fig. 1g), nor the levels of the reduced (Fig. 1c, d) and oxidized (Fig. 1e, f) partners of these cellular redox pairs. During a 60 min incubation a small loss of around 30% and 15% was found for the cellular contents of NADx (Fig. 1a) and of NADPx (Fig. 1b), respectively. This is consistent with data from previous studies on astrocytes and is likely to be caused by the absence of nicotinamide precursors in the buffer used for the incubations [16, 51].

The buffer used for incubations of astrocytes without β-lapachone contained the solvent DMSO in a final concentration of 0.3%. For the parameters investigated and the experimental conditions used (Fig. 1) we have investigated the potential consequences of a DMSO-free incubation for up to 60 min as additional control and compared the data obtained with those shown in Fig. 1 for the 0 µM β-lapachone (0.3% DMSO) condition. The data obtained (3 experiments performed on independently prepared cultures) for the DMSO-containing (shown in Fig. 1) and the DMSO-free condition (data not shown) were almost identical and did not differ significantly.

Presence of β-lapachone in low concentrations of 1 or 3 µM did not affect the contents of NADx (Fig. 1a), NADPx (Fig. 1b) and NAD(P)x (Fig. 1g) compared to the absence of β-lapachone, but caused an immediate oxidation of the reduced nicotinamide cofactors to the respective oxidized redox partners as demonstrated by the complete loss in cellular NADH (Fig. 1c) and NADPH (Fig. 1d) contents.

In addition to the rapid oxidation of NADH (Fig. 1c, e) and NADPH (Fig. 1d, f), application of β-lapachone in concentrations of 10 or 15 µM also caused a rapid, and transient increase in the cellular contents of NADPx (Fig. 1b) and NADP^+^ (Fig. 1f) and a severe and highly significant loss in the cellular contents of NADx (Fig. 1a), NAD^+^ (Fig. 1e) and NAD(P)x (Fig. 1g) within 15 min. None of the conditions applied caused any obvious cell toxicity in the investigated time frame as demonstrated by the absence of a significant increase in extracellular LDH activity (Fig. 1h; Table 1).

**Table 1:** Effects of a treatment of cultured astrocytes with β-lapachone or β-lapachone plus PJ34 on the cellular redox pairs and on the ATP content.

	Initial contents		None		15 µM β-lapachone			15 µM β-lapachone + 3 µM PJ34		
	Specific content (nmol/mg protein)	Number of experiments (independent cultures)	Specific content (nmol/mg protein)	Number of experiments (independent cultures)	Specific content (nmol/mg protein)	Percental content (% of none)	Number of experiments (independent cultures)	Specific content (nmol/mg protein)	Percental content (% of none)	Number of experiments (independent cultures)
NADx content	3.41 ± 0.42***	*35 (27)*	2.68 ± 0.39	*19 (15)*	0.54 ± 0.27***	20	*36 (29)*	2.07 ± 0.58***	77	*18 (15)*
NAD^+^ content	2.53 ± 0.42***	*26 (19)*	1.99 ± 0.57	*15 (11)*	0.38 ± 0.17***	19	*26 (19)*	1.73 ± 0.37	87	*14 (11)*
NADH content	0.79 ± 0.19**	*26 (19)*	0.61 ± 0.24	*15 (11)*	0.09 ± 0.14***	14	*26 (19)*	0.09 ± 0.15***	15	*14 (11)*
NADPx content	0.62 ± 0.08	*28 (21)*	0.59 ± 0.08	*15 (12)*	0.82 ± 0.11***	140	*28 (21)*	0.99 ± 0.21***	168	*20 (15)*
NADP^+^ content	0.38 ± 0.12	*22 (17)*	0.36 ± 0.12	*11 (9)*	0.82 ± 0.12***	229	*22 (17)*	1.09 ± 0.12***	306	*14 (11)*
NADPH content	0.23 ± 0.12	*22 (17)*	0.22 ± 0.10	*11 (9)*	0.01 ± 0.02***	6	*22 (17)*	0.02 ± 0.02***	7	*14 (11)*
NAD(P)x content	4.00 ± 0.43***	*26 (21)*	3.33 ± 0.44	*14 (12)*	1.31 ± 0.24***	39	*26 (21)*	3.09 ± 0.46	93	*18 (15)*
GSx content	56.12 ± 4.65	*10 (8)*	54.35 ± 8.04	*3 (3)*	40.81 ± 4.60**	75	*10 (8)*	42.46 ± 4.99**	78	*7 (5)*
GSSG content (as GSx)	0.28 ± 0.38	*10 (8)*	0.07 ± 0.11	*3 (3)*	27.18 ± 5.75***	40,913	*10 (8)*	28.09 ± 5.81***	42,282	*7 (5)*
GSH content	55.84 ± 4.89	*10 (8)*	54.28 ± 8.14	*3 (3)*	13.63 ± 3.04***	25	*10 (8)*	14.38 ± 4.00***	26	*7 (5)*
ATP content	28.99 ± 2.16	*16 (13)*	29.66 ± 4.18	*6 (6)*	14.83 ± 6.58***	50	*19 (16)*	22.62 ± 7.15*	76	*11 (9)*

Astrocyte cultures were incubated in glucose-containing incubation buffer without or with 15 µM β-lapachone or with 15 µM β-lapachone plus 3 µM of the PARP inhibitor PJ34 for 15 min at 37 °C. After the incubation, the given cellular parameters were determined. The data shown represent the means ± SD of values determined in n independent experiments performed on the given number (in brackets) of independently prepared cultures. The initial protein content of the cultures (determined for 37 experiments on 27 independent cultures) was 147 ± 11 µg/well. The initial LDH activity (determined for 28 experiments on 21 independent cultures) was 157 ± 37 nmol/(min x well). The extracellular LDH activity found after the 15 min incubation accounted for 5 ± 4% (none; 12 experiments on 10 cultures), 4 ± 4% (β-lapachone; 29 experiments on 21 cultures) and 5 ± 5% (β-lapachone + PJ34; 18 experiments on 13 cultures) of the initial cellular LDH activity. The significance of differences (ANOVA with Bonferroni post hoc test) compared to the data obtained for the control incubation (none) is indicated by *p < 0.05, **p < 0.01 and ***p < 0.001

**Fig. 2:**
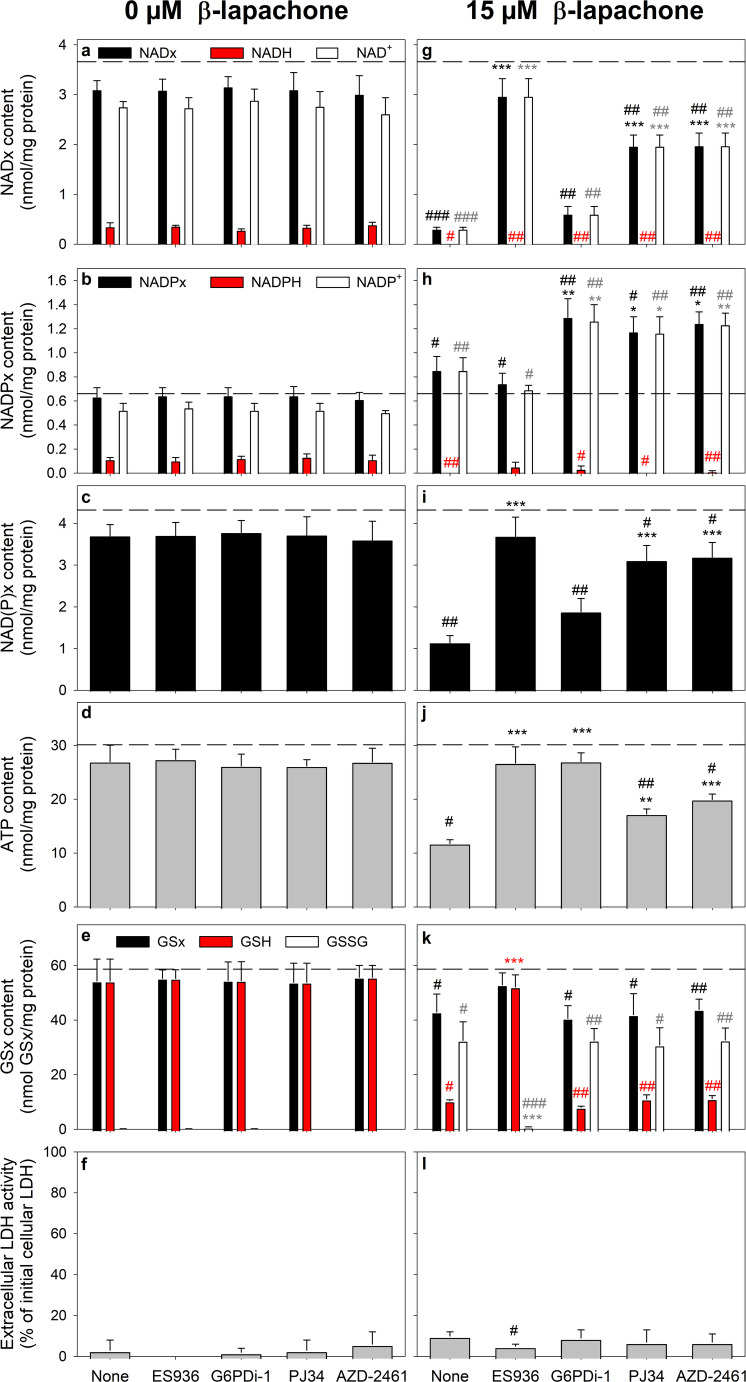
Effects of NQO1-, PPP- and PARP-inhibitors on the cellular redox pairs and on the ATP content of cultured astrocytes that were incubated without or with β-lapachone. The cells were incubated in glucose-containing incubation buffer without (**a**–**f**) or with (**g**–**l**) 15 µM β-lapachone in the absence or the presence of 30 µM ES936, 30 µM G6PDi-1, 3 µM PJ34 or 3 µM AZD-2461. After 15 min of incubation, the cellular contents of NADx (**a**, **g**) and NADPx (**b**, **h**) as well as of the respective reduced and oxidized partners of these redox pairs were determined. In addition, the NAD(P)x content was calculated (**c**, **i**), the specific contents of ATP (**d**, **j**) and GSx (**e**, **k**), were quantified and the extracellular LDH activity was measured (**f**, **l**). The initial NADx content was 3.66 ± 0.39 nmol/mg protein (of which 83 ± 1% represented NAD^+^) and the initial NADPx content was 0.66 ± 0.10 nmol/mg protein (of which 79 ± 0% represented NADP^+^). The initial NAD(P)x content amounted to 4.32 ± 0.48 nmol/mg protein. The initial GSx content was 58.7 ± 1.9 nmol/mg protein (completely consisting of GSH) and the initial ATP content amounted to 30.2 ± 1.0 nmol/mg protein. These initial contents are indicated as dashed lines in the respective panels. The initial LDH activity was 114 ± 9 nmol/(min x well) and the initial protein content of the cultures was 135 ± 11 µg/well. The data shown represent the means ± SD of values determined in three independent experiments performed on independently prepared astrocyte cultures. The significance of differences (ANOVA with Bonferroni post hoc test) compared to the data obtained for the control without inhibitors (none) is indicated by *p < 0.05, **p < 0.01 and ***p < 0.001. The significance of differences (t-test) comparing data of incubations with 0 µM β-lapachone with data from incubations with 15 µM β-lapachone are indicated by ^#^*p* < 0.05, ^##^*p* < 0.01 and ^###^*p* < 0.001

For further investigations on the consequences of β-lapachone-induced oxidative stress on the cellular redox and energy metabolism of astrocytes a 15 min incubation with 15 µM β-lapachone was chosen. For this experimental condition, the specific cellular contents of NADx, NADPx, GSx, of the respective oxidized and reduced redox partners, as well as the specific ATP content and the extracellular LDH activity as indicator for a potential loss in cell viability were determined in a large number of experiments (Table 1). In the absence of β-lapachone, the parameters investigated were hardly affected during a 15 min incubation (Table 1; Fig. 2). At best a 20% decline in the specific cellular NADx content was observed (Table 1) as expected from literature data for such incubations [16, 45]. In contrast, after an incubation of cultured astrocytes with 15 µM β-lapachone for 15 min, 75% of the cellular GSx content accounted for GSSG and the NADx and NADPx contents represented almost exclusively the oxidized partner of each redox pair (Table 1). In addition, the cellular NADx content was found depleted by 80% (Table 1), while the NADPx content was increased by 40% (Table 1). Furthermore, by the β-lapachone treatment the specific cellular ATP content was found lowered to around 50% of the content in control cells, while the cell viability was not compromised by the treatment (Table 1).

NQO1 is required for the reduction of β-lapachone in astrocytes to initiate the β-lapachone-induced oxidative stress [40, 41]. As expected, all the changes that had been caused by a 15 min exposure to 15 µM β-lapachone were prevented by the presence of ES936 (Fig. 2), an inhibitor of NQO1 [40, 41].

The presence of the glucose-6-phosphate inhibitor G6PDi-1 [42] did not affect the parameters investigated for a 15 min incubation in the absence of β-lapachone (Fig. 2a-f), nor did this inhibitor substantially modify the loss in cellular NADx content during a coincubation with 15 µM β-lapachone (Fig. 2g) or the oxidation of cellular GSH (Fig. 2k). However, the presence of G6PDi-1 increased the accumulation of NADPx during the incubation of astrocytes with β-lapachone (Fig. 2h) and prevented the loss in cellular ATP (Fig. 2j).

### PARP-Dependency of the NADx Depletion During β-Lapachone-Induced Oxidative Stress

DNA damage, which is associated with oxidative stress, is known to induce the activation of the NAD^+^-consuming PARP enzymes in various cell types [39, 52, 53], including astrocytes [54, 55]. In order to test whether PARP-catalysed NAD^+^ consumption may contribute to the observed loss in NADx during β-lapachone-induced oxidative stress, cultured astrocytes were exposed to 15 µM β-lapachone in the absence or the presence of the PARP 1–2 inhibitor PJ34 [55, 56] or the PARP 1–3 inhibitor AZD-2461 [57, 58]. In the absence of β-lapachone none of these inhibitors affected the determined metabolic parameters significantly, compared to the respective control without inhibitors (Fig. 2a-e). In contrast, compared to the β-lapachone incubation without PARP inhibitors, the presence of either PJ34 or AZD-2461 lowered the loss in cellular NADx by around 60% (Fig. 2g), doubled the increase in cellular NADPx content (Fig. 2h), almost maintained the initial specific NAD(P)x content (Fig. 2i) and significantly lowered the loss in the cellular ATP content (Fig. 2j). However, the PARP inhibitors did not affect the oxidative stress-induced complete oxidation of NADH and NADPH nor the strong cellular accumulation of GSSG (Fig. 2; Table 1).

### Consequences of PARP-Dependent NADx-Depletion on the Energy Metabolism of Astrocytes

**Fig. 3:**
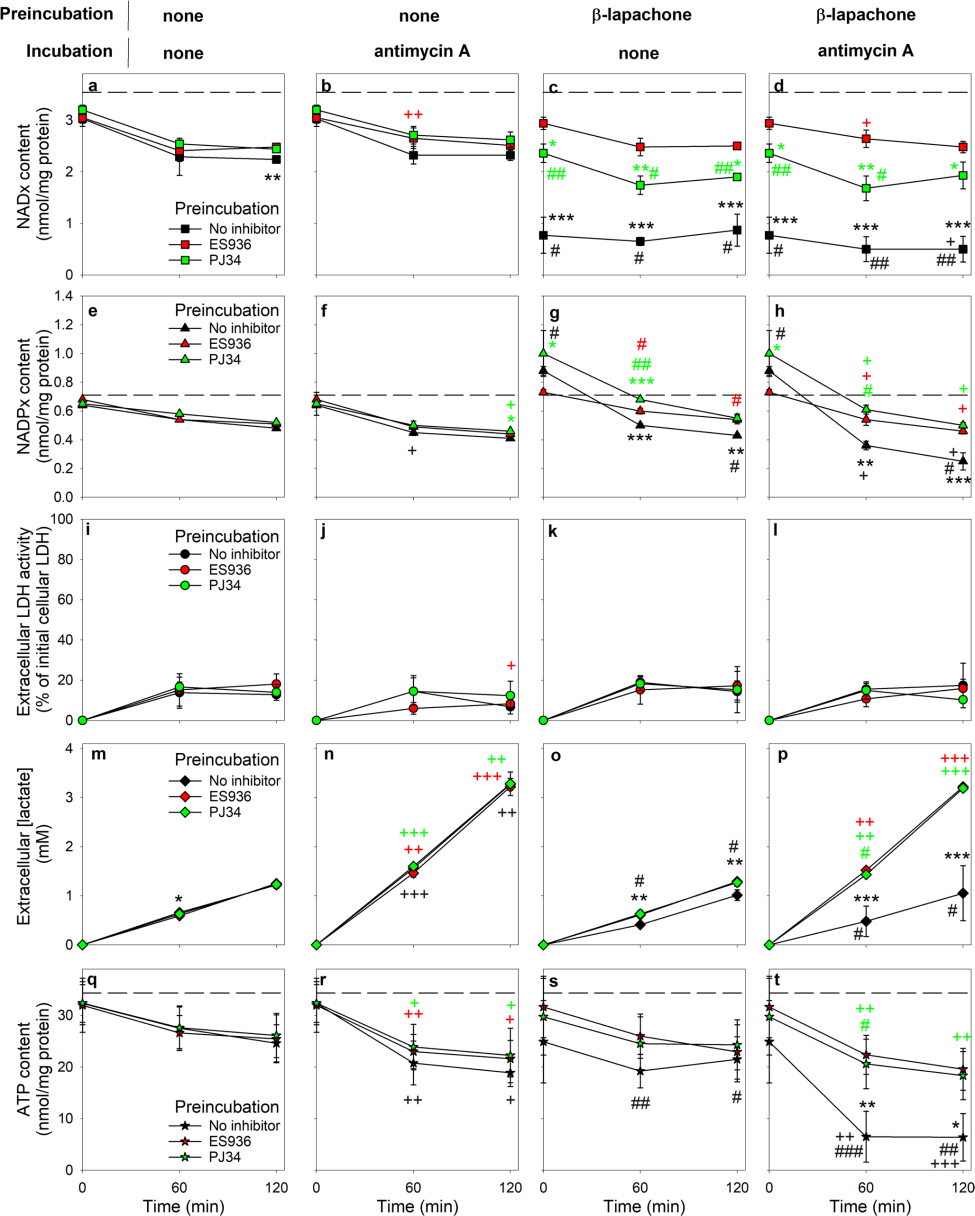
Time-dependent alterations in cellular parameters and lactate formation of cultured astrocytes following β-lapachone-induced oxidative stress. The cells were preincubated in glucose-containing incubation buffer without (none) or with 15 µM β-lapachone in the absence (no inhibitor) or the presence of either 30 µM ES936 or 3 µM PJ34 for 15 min at 37 °C as indicated. After washing the cells, they were incubated for up to 120 min at 37 °C in glucose-containing incubation buffer in the absence or the presence of 1 µM antimycin A. After the preincubation (value at 0 min) and after the following incubations (values at 60–120 min) the cellular contents of NADx (**a**–**d**), NADPx (**e**–**h**), the extracellular LDH activity (**i**–**l**), the extracellular lactate concentration (**m**–**p**) and the cellular ATP content (**q**–**t**) were determined. The initial cellular contents (indicated by dashed lines) amounted to 3.54 ± 0.07 nmol NADx/mg protein, 0.71 ± 0.04 nmol NADPx/mg protein and 34.3 ± 4.7 nmol ATP/mg protein. The initial cellular LDH activity was 178 ± 17 nmol/(min x well) and the initial protein content of the cultures was 155 ± 7 µg/well. The data shown represent the means ± SD of values determined in three independent experiments performed on independently prepared astrocyte cultures. The significance of differences (ANOVA with Bonferroni post hoc test) comparing data of the different preincubation conditions (no inhibitor, PJ34) to the data obtained for the preincubation with ES936 (red symbols) is indicated by *p < 0.05, **p < 0.01 and ***p < 0.001 in the colours used for the conditions. The significance of differences (t-test) for each timepoint compared to the data obtained for the respective control without β-lapachone (^#^*p* < 0.05, ^##^*p* < 0.01 and ^###^*p* < 0.001) or the respective control incubation without antimycin A (^+^*p* < 0.05, ^++^*p* < 0.01 and ^+++^*p* < 0.001) is indicated in the colour of the respective condition

Cultured astrocytes produce substantial amounts of lactate by aerobic glycolysis which is the consequence of efficient regeneration of the NAD^+^ needed for GAPDH-mediated oxidation during glycolysis. This basal astrocytic lactate production is strongly stimulated, if mitochondrial oxidative phosphorylation is impaired [59]. In order to test whether the NADx depletion caused by a 15 min preincubation with β-lapachone may affect glycolytic lactate production and ATP regeneration, cultured astrocytes were preincubated for 15 min with or without β-lapachone in the absence or the presence of the NQO1 inhibitor ES936 or the PARP inhibitor PJ34, washed and then further incubated for up to 120 min with glucose in the absence or the presence of the complex III inhibitor antimycin A. None of the conditions applied caused any obvious loss in membrane integrity in the investigated timeframe as demonstrated by the absence of any significant increase in extracellular LDH activity compared to the respective control conditions (Fig. 3i-l).

For incubations of astrocyte cultures that had not been exposed to β-lapachone, a slight loss of around 20 to 30% was observed for the specific cellular contents of NADx (Fig. 3a, b), NADPx (Fig. 3e, f) and ATP (Fig. 3q, r) during the 2 h incubation in glucose-containing IB both in the absence and the presence of antimycin A. During these incubations lactate was release from the cells proportional with time, reaching extracellular concentrations of around 1.2 mM and 3.3 mM after 2 h for incubations without and with antimycin A, respectively (Fig. 3m, n). All these parameters were hardly affected by the absence or the presence of either the NQO1 inhibitor ES936 or the PARP inhibitor PJ34 during the preincubation (Fig. 3a, b, e, f, m, n, q, r).

After a 15 min preincubation with 15 µM β-lapachone, the contents of the cellular NADx and ATP were found lowered by around 80% (Fig. 3c) and 30% (Fig. 3s), respectively, and these levels remained almost unchanged during a following incubation in the absence of antimycin A, while the specific NADPx content was transiently increased by around 20% compared to the initial cellular level (Fig. 3g). For this condition, the amounts of lactate released from β-lapachone-preincubated cells were slightly but significantly lowered (Fig. 3o) compared to the respective β-lapachone-free control (Fig. 3m). All these alterations compared to the respective control incubations (absence of β-lapachone) were prevented by the presence of the NQO1 inhibitor ES936 during the preincubation (Fig. 3c, g, k, o, s). In addition, the presence of the PARP inhibitor PJ34 during the preincubation with β-lapachone at least partially prevented the loss in cellular NADx (Fig. 3c) and ATP (Fig. 3s) and these levels remained almost unaltered during the subsequent main incubation. Presence of PJ34 during the preincubation with β-lapachone caused a strong increase in the content of NADPx that declined severely during the main incubation (Fig. 3g), while a normal lactate release was maintained (Fig. 3o).

For antimycin A main incubations of astrocytes that had been preincubated with β-lapachone alone, specific contents of NADx (Fig. 3d) and NADPx (Fig. 3h) did only slightly differ to the respective values of the antimycin A-free main incubations (Fig. 3c, g). However, these cells were unable to increase their glycolytic lactate release in the presence of antimycin A (Fig. 3p) and suffered from a severe further loss in the cellular ATP content (Fig. 3t). These adverse effects of an antimycin A treatment during the main incubation were completely prevented, when the cells had been preincubated with β-lapachone in the presence of either the NQO1 inhibitor ES936 or the PARP inhibitor PJ34 (Fig. 3p, t) and therefore had maintained a higher cellular NADx content (Fig. 3d).

### Restoration of the Initial Cellular Contents of Redox Cofactors and ATP Following β-Lapachone-Induced Oxidative Stress

**Fig. 4:**
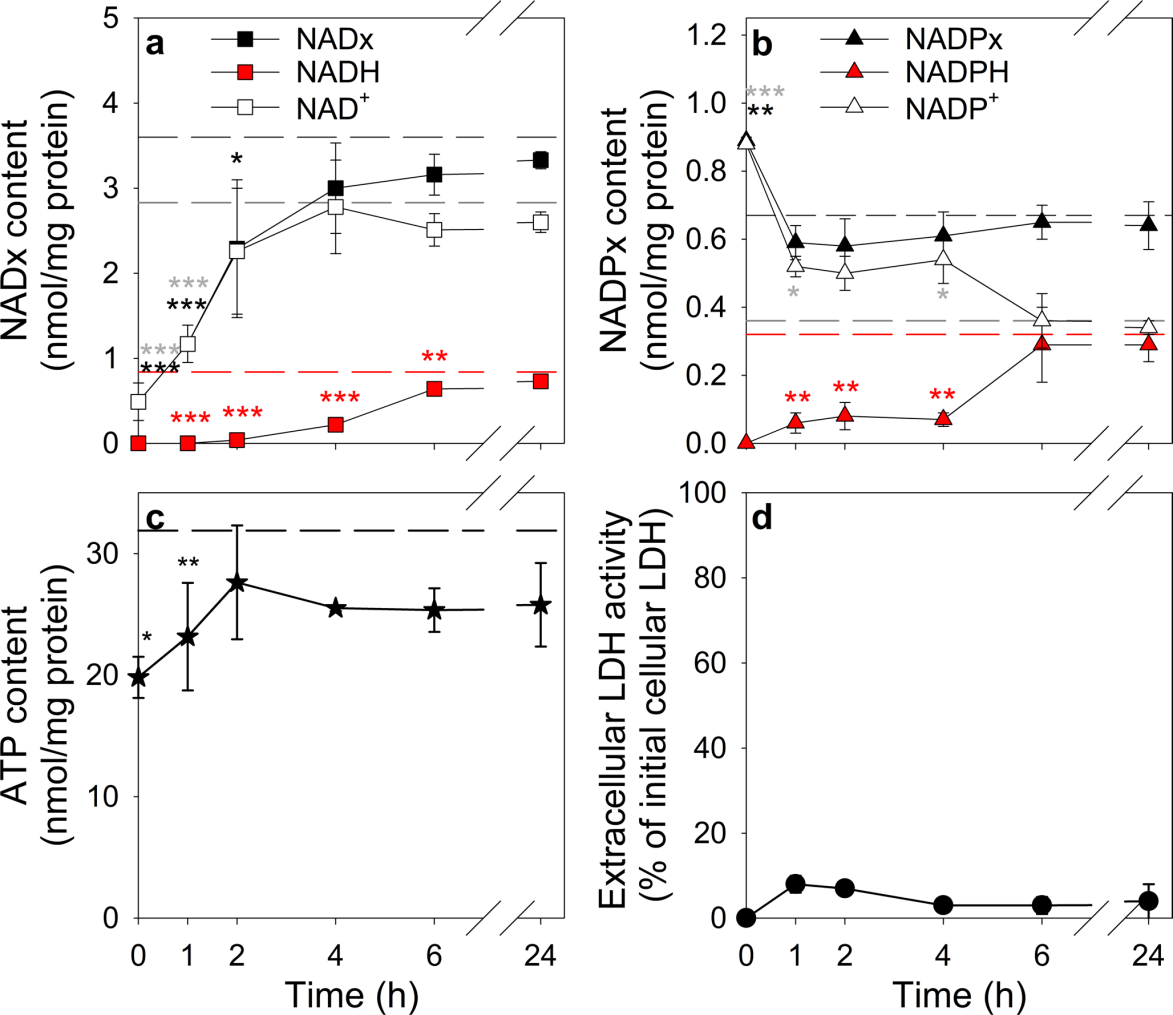
Test for restoration of cellular nicotinamide cofactors and ATP following β-lapachone-induced oxidative stress. The cells were preincubated in glucose-containing incubation buffer in the presence of 15 µM β-lapachone for 15 min at 37 °C. After the preincubation, the cells were washed twice with DMEM followed by an incubation with serum-containing culture medium in an incubator with 10% CO_2_ supply for the given time points before cellular contents of nicotinamide cofactors (**a**, **b**) and ATP (**c**) as well as the extracellular LDH activity (**d**) were determined. The initial contents of the parameters determined are indicated by the dashed lines in the panels a-c and represent 3.60 ± 0.52 nmol NADx/mg protein (of which 79 ± 8% represented NAD^+^), 0.67 ± 0.05 nmol NADPx/mg protein (of which 54 ± 18% represented NADP^+^) and 31.9 ± 2.3 nmol ATP/mg protein. The initial protein content of the cultures was 141 ± 6 µg/well and the initial LDH activity was 122 ± 8 nmol/(min x well). The data shown represent the means ± SD of values determined in three independent experiments performed on independently prepared primary cultures. The significance of differences (ANOVA with Bonferroni post hoc test) compared to the data obtained for the initial content (black, red and grey lines) is indicated by *p < 0.05, **p < 0.01 and ***p < 0.001) in the colour used for the symbols indicating the respective contents

To study the ability of cultured astrocytes to reestablish their initial cellular contents of NADx, NADPx and ATP after oxidative stress, the cells were preincubated with 15 µM β-lapachone for 15 min, washed and incubated for up to 24 h (recovery period) in serum-containing culture medium. This incubation did not cause any obvious cell damage as indicated by the low extracellular LDH activity that was detectable during the recovery incubation (Fig. 4d). The exposure of cultured astrocytes to β-lapachone-induced oxidative stress for 15 min severely lowered the NADx and ATP contents and elevated the cellular NADPx content (Fig. 4) as shown before (Table 1). However, within 2 h of the incubation in culture medium the cells restored 80% of the initial ATP content (Fig. 4c) and after 4 h around 90% of their initial NAD^+^ content (Fig. 4a) to levels that did not significantly differ to the initial cellular contents (black lines) and they remained at these high levels for up to 24 h (Fig. 4a, c). The elevated NADPx content found after the oxidative stress phase was lowered to values slightly below the initial content within the first 1 h of the recovery period and the determined cellular NADPx content remained similar to the initial NADPx content for the further incubation time of up to 24 h (Fig. 4b).

Analysis of the individual contents of the reduced and oxidized partners of the NADx and NADPx redox pairs revealed that the high content of the oxidized partners determined after the preincubation was maintained for some hours and that it took around 6 h before the cellular contents of NADH and NADPH had increased to the levels found for untreated cells (Fig. 4a, b).

**Fig. 5:**
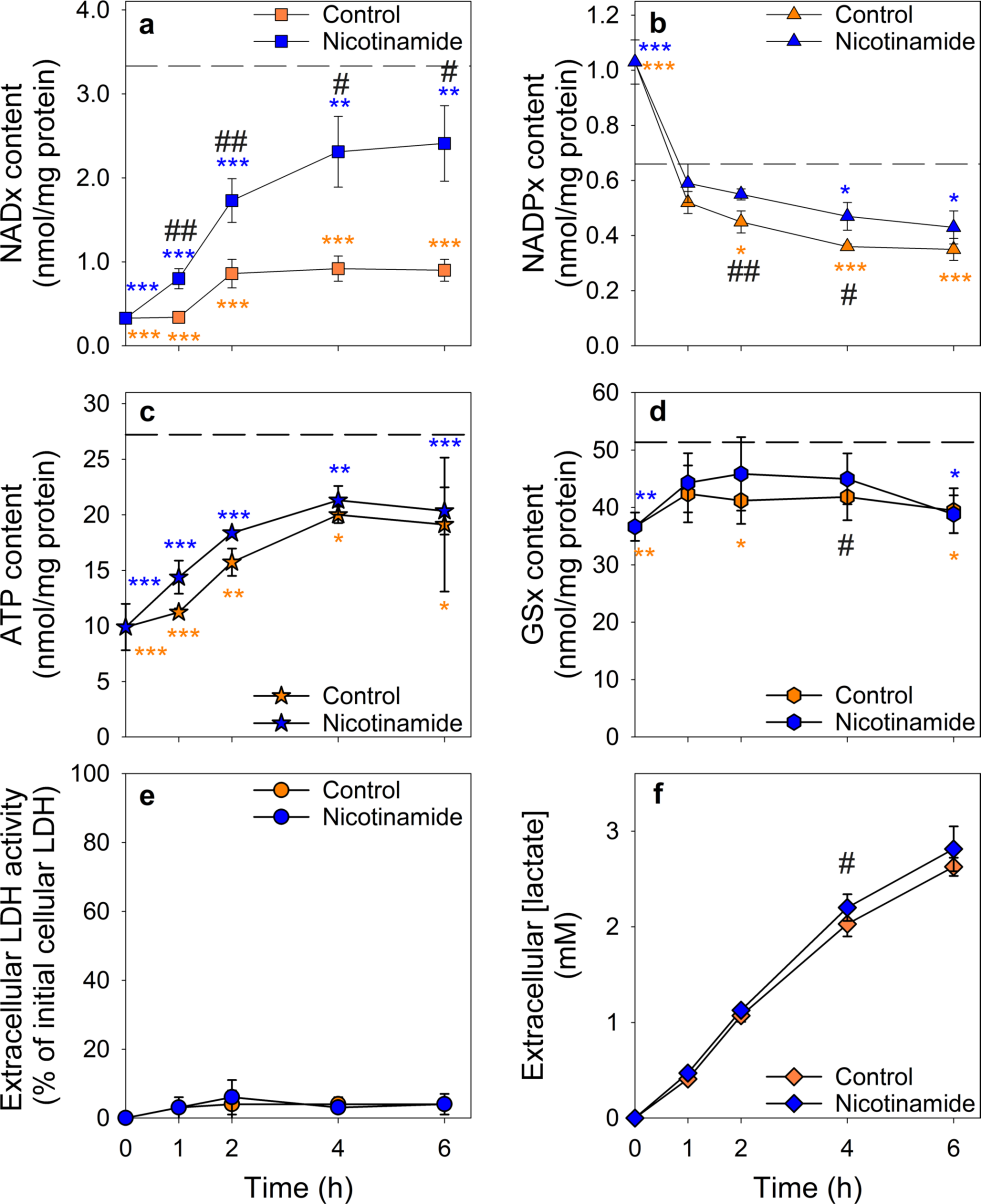
Test for effects of nicotinamide on the restoration of cellular redox cofactors and ATP following β-lapachone-induced oxidative stress. The cells were preincubated in glucose-containing incubation buffer in the presence of 15 µM β-lapachone for 15 min at 37 °C, washed twice with IB, and incubated with glucose-containing incubation buffer in the absence (control) or the presence of 1 mM nicotinamide. For the given time points, the cellular contents of nicotinamide cofactors (**a**, **b**), ATP (**c**) and GSx (**d**) as well as the extracellular LDH activity (**e**) and the extracellular lactate concentration (**f**) were determined. The initial contents of the parameters determined are indicated by the dashed lines in the panels a-d and represent 3.33 ± 0.05 nmol NADx/mg protein, 0.66 ± 0.14 nmol NADPx/mg protein, 27.2 ± 0.5 nmol ATP/mg protein and 51.4 ± 3.6 nmol GSx/mg protein. The initial protein content of the investigated cultures was 141 ± 15 µg/well and the initial LDH activity was 130 ± 19 nmol/(min x well). The data shown represent the means ± SD of values determined in three independent experiments performed on independently prepared primary cultures. The significance of differences (ANOVA with Bonferroni post hoc test) compared to the data obtained for the initial content (black dashed lines) is indicated by *p < 0.05, **p < 0.01 and ***p < 0.001 in the colours of the symbols representing the respective conditions. The significance of differences (t-test) comparing data from incubations without to those with nicotinamide are indicated by ^#^*p* < 0.05 and ^##^*p* < 0.01

A nicotinamide precursor has been shown to be required for efficient NAD^+^ synthesis [60]. To test for the ability of nicotinamide to foster efficient cellular NAD^+^ restoration following oxidative stress, astrocytes were preincubated with 15 µM β-lapachone for 15 min and then incubated in glucose-containing incubation buffer in the absence or the presence of nicotinamide. In the absence of nicotinamide, the cellular NADx content slightly increased to around 1 nmol/mg protein within the first two hours of the recovery phase, which represents around 30% of the initial cellular content, and remained at this level during longer incubations (Fig. 5a). During the recovery period following oxidative stress, the elevated cellular NADPx content strongly declined within the first hour of the recovery period and slightly continued to decline further during longer incubations (Fig. 5b). In contrast, the cellular values for GSx remained almost unaltered during the recovery phase (Fig. 5d), the viability was not compromised (Fig. 5e) and the cells released lactate almost proportional to time resulting in an extracellular concentration of around 2.7 mM after 6 h of incubation (Fig. 5f).

Presence of nicotinamide during the recovery of astrocytes following β-lapachone-induced oxidative stress strongly accelerated and increased the NADx restoration and the cellular content reached around 80% of the initial values after 4 h (Fig. 5a). Presence of nicotinamide also decreased the cellular loss in NADPx compared to the control incubation (Fig. 5b), while at best small effects were observed for a nicotinamide-treatment regarding the ATP restoration (Fig. 5c), the cellular GSx content (Fig. 5d), the cell viability (Fig. 5e) or the cellular lactate production (Fig. 5f).

Analysis of the individual contents of the reduced and oxidized partners of the three redox pairs revealed that for the GSH and GSSG redox pair, the high ratio of GSH to GSSG of untreated cells had been fully reestablished within 1 h of the recovery phase (Table 2). In contrast, even after 1 h of recovery the reduced partners, NADH and NADPH, accounted for less than 10% of the cellular NADx and NADPx redox pairs and it required a 6 h recovery phase before substantial amounts of NADH and NADPH were detectable in the cells. These alterations in the contents of the three redox pairs were not affected by the absence or the presence of nicotinamide during the recovery phase following β-lapachone-induced oxidative stress (Table 2).

**Table 2:** Effects of the application of nicotinamide on the cellular redox pairs during the restoration phase following β-lapachone-induced oxidative stress.

	Initial contents		15 µM β-lapachone preincubation		Control 1 h incubation		Nicotinamide 1 h incubation		Control 6 h incubation		Nicotinamide 6 h incubation	
	Specific content (nmol/mg protein)	(%)	Specific content (nmol/mg protein)	(%)	Specific content (nmol/mg protein)	(%)	Specific content (nmol/mg protein)	(%)	Specific content (nmol/mg protein)	(%)	Specific content (nmol/mg protein)	(%)
NADx content	3.33 ± 0.05	100	0.33 ± 0.07***	100	0.34 ± 0.05***	100	0.80 ± 0.12***^,##^	100	0.90 ± 0.13***	100	2.41 ± 0.45**^,#^	100
NAD^+^ content	2.39 ± 0.11	72 ± 2	0.33 ± 0.07***	100 ± 1**	0.32 ± 0.02***	95 ± 9**	0.78 ± 0.10***^,#^	98 ± 2***	0.75 ± 0.04***	84 ± 10	2.09 ± 0.04^#^	87 ± 4***
NADH content	0.94 ± 0.06	28 ± 2	0.00 ± 0.00***	0 ± 1**	0.02 ± 0.03***	5 ± 9**	0.02 ± 0.02***	2 ± 2***	0.15 ± 0.12***	16 ± 10	0.33 ± 0.15***	13 ± 4***
NADPx content	0.66 ± 0.14	100	1.03 ± 0.08**	100	0.52 ± 0.04	100	0.59 ± 0.07	100	0.35 ± 0.04**	100	0.43 ± 0.11*	100
NADP^+^ content	0.26 ± 0.13	63 ± 13	1.02 ± 0.08***	99 ± 0***	0.45 ± 0.01*	87 ± 4***	0.53 ± 0.06**	89 ± 4***	0.23 ± 0.03	65 ± 10*	0.82 ± 0.06	63 ± 6*
NADPH content	0.41 ± 0.02	37 ± 13	0.01 ± 0.00***	1 ± 0***	0.07 ± 0.02***	13 ± 4***	0.06 ± 0.03***	11 ± 4***	0.12 ± 0.05***	35 ± 10*	0.18 ± 0.05***	37 ± 6*
GSx content	51.36 ± 3.55	100	36.64 ± 2.48**	100	42.36 ± 4.95	100	44.29 ± 5.14	100	39.45 ± 3.90*	100	38.80 ± 3.29*	100
GSSG content (as GSx)	0.80 ± 0.03	2 ± 0	23.15 ± 3.09***	63 ± 5***	1.05 ± 0.20	3 ± 1	0.87 ± 0.08	2 ± 0	0.87 ± 0.08	2 ± 0	0.65 ± 0.06	2 ± 0
GSH content	50.56 ± 3.52	98 ± 0	13.49 ± 1.46***	37 ± 5***	41.31 ± 5.12*	97 ± 1	43.42 ± 5.06	98 ± 0	38.58 ± 3.83*	98 ± 0	38.14 ± 3.34**	98 ± 0

Astrocyte cultures were preincubated in glucose-containing incubation buffer with 15 µM β-lapachone for 15 min at 37 °C. After washing, the cells were incubated for 1 or 6 h in glucose-containing incubation buffer in the absence (control) or the presence of 1 mM nicotinamide before the given cellular parameters were determined. The initial protein content of the cultures was 141 ± 15 µg/well. The data shown represent the means ± SD of values determined in three independent experiments performed on independently prepared primary cultures (see Fig. 5). The percental contents (%) were calculated by dividing the content of the oxidized or reduced species of each redox pair by the respective total content. The significance of differences (ANOVA with Bonferroni post hoc test) of data for either without or with nicotinamide at the different time points compared to the initial contents is indicated by *p < 0.05, **p < 0.01 and ***p < 0.001. The significance of differences (t-test) comparing data of incubations without and with nicotinamide are indicated by ^#^*p* < 0.05 and ^##^*p* < 0.01

**Fig. 6:**
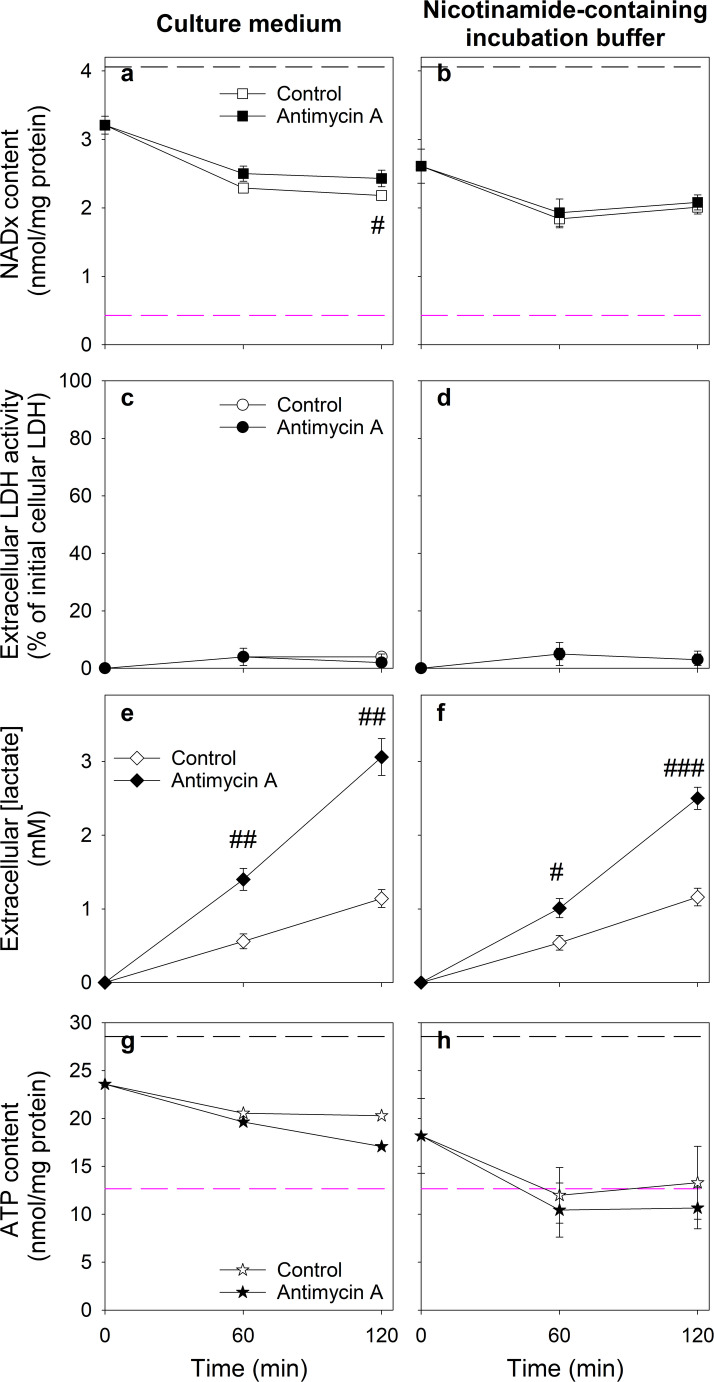
Consequences of a restoration phase after β-lapachone-induced oxidative stress on the lactate formation by cultured astrocytes. The cells were preincubated in glucose-containing incubation buffer in the presence of 15 µM β-lapachone for 15 min at 37 °C. Then, the cells were incubated for a 24 h recovery phase at 37 °C in either culture medium (with CO_2_ supply) or incubation medium (incubation buffer with 5 mM glucose and 1 mM nicotinamide; without CO_2_ supply). Finally, the cells were incubated for up to 120 min at 37 °C in glucose-containing incubation buffer in the absence (control) or the presence of 1 µM antimycin A. After the preincubation, the recovery phase and after the final incubations (values at 60–120 min) the cellular contents of NADx (**a**, **b**), the extracellular LDH activity (**c**, **d**), the extracellular lactate concentration (**e**, **f**) and the cellular ATP content (**g**, **h**) were determined. The initial cellular contents (indicated by the black dashed lines) amounted to 4.06 ± 0.22 nmol NADx/mg protein and 28.6 ± 1.2 nmol ATP/mg protein and the cellular contents after the 15 min oxidative stress phase (indicated by the pink dashed lines) amounted to 0.43 ± 0.07 nmol NADx/mg protein and 12.7 ± 1.9 nmol ATP/mg protein. The initial cellular LDH activity was 189 ± 24 nmol/(min x well) and the initial protein content of the cultures was 154 ± 7 µg/well. The data shown represent the means ± SD of values determined in three independent experiments performed on independently prepared astrocyte cultures. The significance of differences (t-test) for each time point comparing data obtained for incubations without (control) or with antimycin A is indicated by ^#^*p* < 0.05, ^##^*p* < 0.01 and ^###^*p* < 0.001)

To investigate whether astrocytes that had restored their cellular NADx content were able to regain their ability to upregulate glycolytic lactate production during an exposure to antimycin A, cultured astrocytes were preincubated for 15 min with 15 µM β-lapachone followed by a 24 h restoration phase in either culture medium or glucose-containing incubation buffer with 1 mM nicotinamide. By the preincubation with β-lapachone, the cellular NADx and ATP levels had been lowered by around 90% and 50%, respectively (Fig. 6), but after the 24 h recovery phase the contents were restored to around 70–80% of the initial specific contents in both media (Fig. 6a, b, g, h). In addition, after the recovery in both media cultured astrocytes were able to increase the lactate production and release during exposure to antimycin A. The detectable extracellular lactate concentrations after 2 h of incubation was increased by antimycin A exposure from 1.14 to 3.06 mM for cells that had recovered in culture medium and from 1.16 to 2.50 mM for cells recovered in nicotinamide-containing incubation buffer (Fig. 6e, f), similar to values found for untreated cells (Fig. 3m, n).

## Discussion

A short-time exposure to β-lapachone-induced oxidative stress was used as experimental paradigm to investigate alterations in the interplay between the different redox pairs during oxidative stress and potential adverse consequences for the energy metabolism of cultured astrocytes. The basal specific contents of untreated cultured astrocytes of GSx, NADx, NADPx, the respective ratios of oxidized to reduced partner of each redox pair, as well as the specific ATP content (Table 1) were in the range of data recently reported and discussed for cultured astrocytes [16, 34].

The treatment with 15 µM β-lapachone for 15 min led to a substantial accumulation of GSSG, an increase in the NADPx content and a loss in the cellular NADx and ATP contents (Table 1; Fig. 2). In addition, the treated astrocytes had lost their ability to upregulate glycolytic lactate production during a subsequent incubation with the respiratory chain inhibitor antimycin A (Fig. 3). All these consequences of the β-lapachone-treatment were prevented by the additional presence of the NQO1 inhibitor ES936 (Fig. 2) [41], demonstrating that the effects observed were caused by β-lapachone-induced oxidative stress [39] and not by other potential β-lapachone effects [35, 37].

Exposure of cultured astrocytes to β-lapachone induced a rapid oxidation of cellular NADH and NADPH, even with low β-lapachone concentrations of 1 or 3 µM (Fig. 1). This is consistent with the ability of such low concentrations of β-lapachone to foster superoxide-dependent WST1 reduction [41] but might also be a consequence of a direct oxidation of NAD(P)H by the reactive oxygen species generated [61]. However, GSSG accumulation in astrocytes was not observed for treatments with β-lapachone in concentrations below 5 µM [41], most likely due to the high capacity of astrocytes to quickly reduce the GSSG that has been generated during the stress phase [11, 16, 40].

**Fig. 7:**
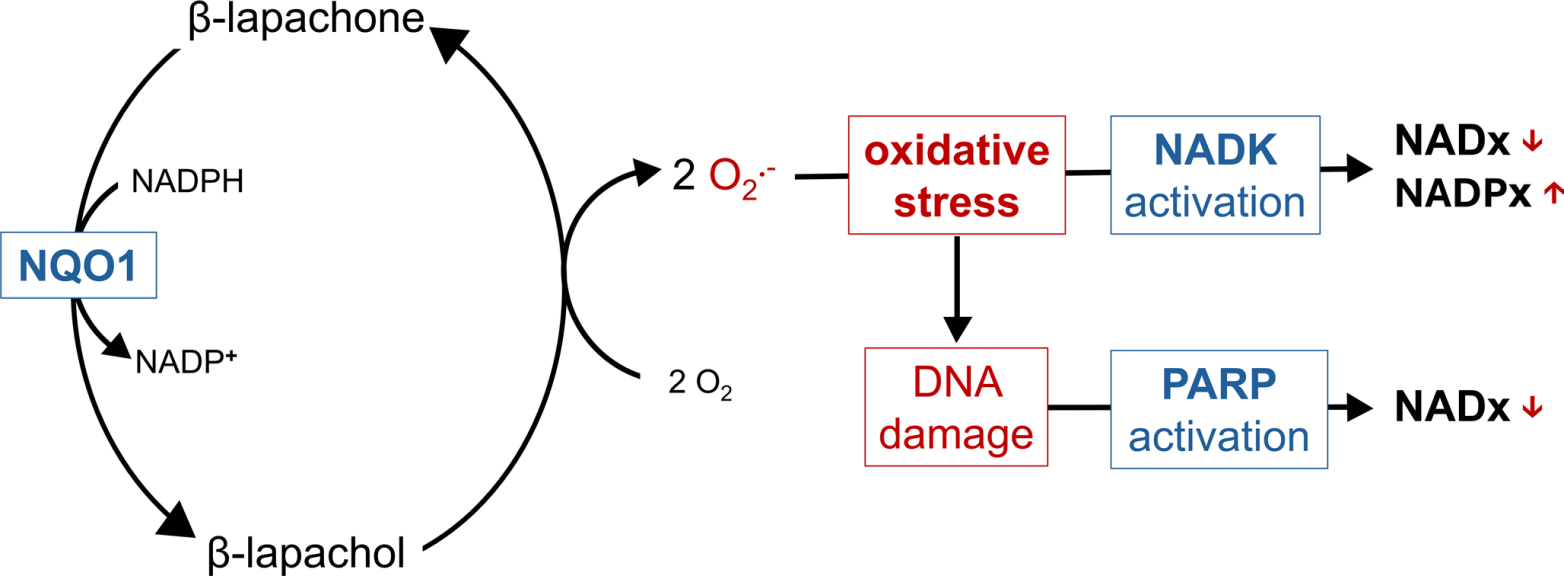
Consequences of β-lapachone-induced oxidative stress on the cellular NADx and NADPx contents of astrocytes. The NADPH-dependent reduction of β-lapachone by NQO1 generates the instable β-lapachol that is oxidized back to β-lapachone in the presence of oxygen, thereby generating superoxide ions which subsequently cause oxidative stress and DNA damage. As consequences, NADK and PARPs are activated which lower the cellular NADx pool by phosphorylation of NAD^+^ to NADP^+^ and by consuming NAD^+^ for ADP-ribosylation, respectively. Accordingly, β-lapachone-induced oxidative stress lowers the cellular NADx pool and increases the cellular NADPx pool, thereby affecting cellular pathways that rely on the presence of the nicotinamide cosubstrates

Severe β-lapachone-induced oxidative stress caused a time- and concentration-dependent loss in the total cellular NADx content (Fig. 1). Most of the loss of NADx during a β-lapachone exposure of astrocytes was prevented by the presence of PARP inhibitors (Table 1; Fig. 2), suggesting that these enzymes are responsible for the majority of the NADx loss observed (Fig. 7). This is in accordance with oxidative stress being known to induce DNA damage that causes a strong activation of PARPs in different cell types [39, 52, 53]. A severe loss of astrocytic NADx after PARP activation has previously been shown for chemical-induced DNA damage [54, 55] and was also described for β-lapachone-induced oxidative stress in cancer cells [38, 39].

Application of 15 µM β-lapachone also caused a transient increase in the total cellular NADPx content (Fig. 1; Table 1), which may in part also contribute to the NADx loss found for β-lapachone-treated astrocytes. This increase in the cellular NADPx content is most likely caused by oxidative stress-induced activation of NADK (Fig. 7) as recently shown for peroxide-treated astrocytes [16] and might support NADPH-dependent antioxidative processes. The increase in cellular NADPx after β-lapachone exposure was even stronger in the presence of PARP inhibitors (Table 1), most likely due to the inhibitor-induced maintenance of a higher cellular NAD^+^ content that was then available as substrate for NADK. With a K_M_ value of astrocytic NADK of 1.3 mM for NAD^+^ [16] and a calculated normal cytosolic concentration of NAD^+^ of around 0.7 mM [51], the NAD^+^ phosphorylating activity of NADK will be proportional to the cellular concentration of NAD^+^, explaining the increased NADPx accumulation when higher cellular NAD^+^ levels are maintained in the presence of PARP inhibitors. However, an elevated NADPx level was also found for astrocytes that had been exposed to β-lapachone in the presence of the G6PDH inhibitor G6PDi-1 (Fig. 2), despite a severely lowered NADx content, which remains to be explained.

An additional consequence of β-lapachone-induced oxidative stress was a decreased cellular ATP content (Table 1). This is most likely a consequence of a preferred use of glucose-6-phosphate for the oxidative part of the PPP, which has been reported to be strongly activated by oxidative stress [62], slowing down the glycolytic flux. This view is supported by the observation that the ATP loss during a 15 min incubation with β-lapachone was completely prevented in the presence of the PPP inhibitor G6PDi-1 (Fig. 2).

The β-lapachone-induced oxidative stress lowered the subsequent lactate production (Fig. 3). Further, it impaired the ability of the cells to upregulate their glycolytic lactate production and to maintain a high ATP content in the presence of antimycin A (Fig. 3). These effects might be a consequence of the severe loss of cellular NADx by oxidative stress-induced PARP activation. An impairment of metabolic enzymes directly by oxidative stress, as reported for the cytosolic GAPDH [63] and the mitochondrial aconitase [64] in astrocytes, appears not to be involved in the observed inability of astrocytes to upregulate the glycolytic flux in the presence of antimycin A, as glycolysis was found upregulated in stressed cells in the presence of the PARP inhibitor PJ34. This condition decreased the cellular NADx loss but did not affect the level of oxidative stress, showing that already 75% of the initial cellular NADx pool were sufficient to allow the antimycin A-induced upregulation of glycolytic lactate production.

After removal of oxidative stress, cultured astrocytes were able to recover from the β-lapachone-induced oxidative stress during incubation in culture medium and reestablished their initial cellular NADx and ATP contents within a few hours (Fig. 4). Thus, all components needed by the cells for maintaining and restoring NADx and ATP are provided to the cells by the applied culture medium. This is consistent with literature data on the maintenance of cellular levels of NADx [51] and ATP [65]. Restoration of cellular ATP has been reported for ATP deprived astrocytes that were treated in the presence of glucose as exclusive extracellular substrate, demonstrating that these cells are able to use endogenous precursors for ATP restoration [34, 45, 65]. In contrast, NADx restoration was rather low in glucose-containing incubation buffer, but strongly increased by the application of nicotinamide (Table 2; Fig. 5), consistent with previous reports showing the need of cultured astrocytes for a NADx precursor like nicotinamide to maintain a high NADx content [51]. However, this presence of nicotinamide did not improve ATP restoration nor the lactate formation, demonstrating that already a low NADx content of around 20% of the normal content is sufficient for ATP restoration and glycolysis as long as the mitochondrial metabolism is intact. After restoration of the cellular NADx levels, efficient glycolytic lactate formation was observed. This is consistent with literature data reporting the reestablishment of glycolytic flux in mouse astrocytes after NADx restoration following a PARP-dependent NADx depletion by exposure to a DNA alkylating substance [66]. The restoration of the cellular NADx content in the recovery period was accompanied by a recovery of the ability to upregulate glycolytic flux in the presence of antimycin A (Fig. 6). This supports the view that a high cellular NADx content is needed for the upregulation. New protein synthesis does not seem to be required to restore the metabolic functions, as a functional recovery was possible in the presence of nicotinamide even in amino acid-deprived incubation medium.

Unexpectedly, after β-lapachone-induced oxidative stress the reestablishment of the initial ratio of the reduced to oxidized partner of the nicotinamide coenzymes took more than 4 h both in culture medium and in glucose-containing buffer, while the reestablishment of the initial high GSH to GSSG content was very rapid and the initial ratio had been fully reestablished within minutes (data not shown; [40]). The delayed restoration of a normal ratio of reduced to oxidized nicotinamide coenzymes suggests that some processes involved in establishing the normal ratio of oxidized to reduced nicotinamide coenzymes were impaired by the stress condition applied. Further studies are required to explore which enzymes and metabolic pathways are involved in the regeneration and/or consumption of NADH and NADPH that may have been affected under the conditions used.

In conclusion, the results presented show that β-lapachone-induced radical formation in astrocytes leads to the activation of PARPs and NADK via oxidative stress (Fig. 7). Such enzyme activations can severely deplete the cellular NADx content in astrocytes affecting ATP regeneration. Thus, the complete chain of events that connects oxidative stress in astrocytes to certain enzyme activations and subsequently to metabolite depletion and metabolic impairment could be analysed by the β-lapachone stress model applied (Fig. 7). So far, only parts of this chain of events have been reported (see discussion above). However, our study required the induction of severe oxidative stress by application of 15 µM β-lapachone for 15 min. For comparison, transient oxidative stress induced by application of H_2_O_2_ plus an inhibitor of PPP also caused an activation of NADK [16], but this treatment seemed not to lead to PARP activation. At least the loss found for the cellular NADx pool in this model of oxidative stress did not exceed the NADK-induced increase in the cellular NADPx pool [16]. Further research is now required to investigate which additional factors define the characteristics and the extend of the astrocytic responses to different types of oxidative stress.

Despite strong responses to β-lapachone-induced oxidative stress, cultured astrocytes have the potential to fully recover from a short period of extensive oxidative stress but need nicotinamide as substrate for efficient NADx restoration. These data provide new insights on the important interplay between the different main redox pairs during and after oxidative stress and on subsequent consequences of such a stress on the astrocytic metabolism while also demonstrating the high potential of astrocytes to recover after severe oxidative stress.

Alterations in the levels and the ratios of nicotinamide cofactors have been connected with various neurological and neurodegenerative disorders as well as with aging [13–15]. Very recently it was even reported that restoration of NAD homeostasis reverses Alzheimer´s pathologies in mice [67]. Thus, more knowledge on the homeostasis of the three cellular redox systems as well as on the effects of oxidative stress on the cellular metabolism is highly warranted to better understand consequences of alterations of such parameters in the normal and diseased brain.

## Data Availability

Enquieries on original data should be directed to the corresponding author.
